# Ideação suicida entre trabalhadores(as) urbanos de um município de grande porte, Bahia, Brasil

**DOI:** 10.1590/0102-311XPT100024

**Published:** 2026-02-13

**Authors:** Tarciso de Figueiredo Palma, Jules Ramon Brito Teixeira, Tânia Maria de Araújo

**Affiliations:** 1 Universidade Estadual de Feira de Santana, Feira de Santana, Brasil.

**Keywords:** Ideação Suicida, Suicídio, Saúde Ocupacional, Estresse Ocupacional, Modelagem de Equações Estruturais, Suicide Ideation, Suicide, Occupational Health, Occupational Stress, Structural Equation Modeling, Ideación Suicida, Suicidio, Salud Laboral, Estrés Laboral, Modelado de Ecuaciones Estructurales

## Abstract

O objetivo foi investigar a associação entre aspectos psicossociais do trabalho, fatores socioeconômicos e a insatisfação global com a ideação suicida em trabalhadores(as), de município baiano de grande porte. Realizou-se estudo transversal, com dados da segunda onda de uma coorte prospectiva da população com 15 anos ou mais de idade, residente na zona urbana de município de grande porte da Bahia, Brasil. Aplicou-se questionário estruturado, incluindo dados sociodemográficos, do trabalho e prática de atividades de lazer. Os estressores ocupacionais foram investigados pelo *Effort-Reward Imbalance* e *Job Content Questionnaire*; a insatisfação global foi mensurada por indicadores do WHOQOL-BREF; e a ideação suicida pelo item 17 do *Self-Reporting Questionnaire*. Foi processada modelagem de equações estruturais, com ponderação para amostras complexas. A frequência de ideação suicida, na amostra de 1.629 trabalhadores(as) estudados, foi de 5%. Efeitos diretos fortes, significantes e com direção para ideação suicida foram observados para insatisfação global (β = 0,72) e com efeito médio para a alta demanda emocional do trabalho (β = 0,27). Sexo feminino, menor renda mensal, ausência de atividades de lazer, comprometimento excessivo com o trabalho e alta demanda emocional apresentaram efeitos indiretos significantes mediados pela insatisfação global. Os caminhos indicados por este estudo possibilitam a compreensão de uma cadeia de acontecimentos que, mediadas por sentimentos de insatisfação, vulnerabiliza o trabalhador e o faz padecer. Esse conjunto de fatores potencializa a ideação suicida e o faz considerar o suicídio como a alternativa que cessará seu sofrimento.

## Abstract (en)

This study aimed to investigate the association between psychosocial aspects of work, socioeconomic factors, and global dissatisfaction with suicidal ideation in workers in a large municipality. This cross-sectional study was carried out with data from the second wave of a prospective cohort with a population aged 15 years or older who lived in the urban area of a large municipality in Bahia, Brazil. A structured questionnaire on sociodemographic, work, and leisure data was applied. Occupational stressors were investigated by the *Effort-Reward Imbalance* and *Job Content Questionnaire*, global dissatisfaction was measured by WHOQOL-BREF indicators, and suicidal ideation was evaluated by item 17 of the *Self-Reporting Questionnaire*. Structural equation modeling was processed and complex samples were weighted. Ideation frequency in the 1,629 studied workers totaled 5%, showing strong, significant, and directed direct effects with global dissatisfaction (β = 0.72) and a medium one for the high emotional demand of work (β = 0.27). The female gender, lower monthly income, absence of leisure activities, excessive commitment to work, and high emotional demand showed significant indirect effects (mediated by global dissatisfaction). The paths in this study evince a chain of events that, mediated by feelings of dissatisfaction, make workers vulnerable and make them suffer. This set of factors potentiates suicidal ideation and makes them consider suicide as an alternative that will stop their suffering.

## Abstract (es)

El objetivo fue investigar la asociación entre aspectos psicosociales del trabajo, factores socioeconómicos y la insatisfacción general con las ideas suicidas en trabajadores y trabajadoras de un municipio de gran tamaño. Se realizó un estudio transversal con datos de la segunda oleada de una cohorte prospectiva de la población mayor de 15 años residente en la zona urbana de un municipio de gran tamaño de Bahía, Brasil. Se aplicó un cuestionario estructurado que incluía datos sociodemográficos, laborales y sobre la práctica de actividades de ocio. Los factores estresantes ocupacionales se investigaron mediante el *Effort-Reward Imbalance* y el *Job Content Questionnaire*; la insatisfacción general se midió mediante indicadores del WHOQOL-BREF; y las ideas suicidas, mediante el ítem 17 del *Self-Reporting Questionnaire*. Se procesó el modelado de ecuaciones estructurales, con ponderación para muestras complejas. La frecuencia de las ideas suicidas en la muestra de 1.629 trabajadores y trabajadoras en estudio fue del 5%. Se observaron efectos directos fuertes, significativos y orientados hacia la ideas suicidas en la insatisfacción general (β = 0,72) y un efecto medio en la alta demanda emocional del trabajo (β = 0,27). El sexo femenino, los ingresos mensuales más bajos, la ausencia de actividades de ocio, el compromiso excesivo con el trabajo y la alta demanda emocional presentaron efectos indirectos significativos mediados por la insatisfacción general. Las vías indicadas por este estudio permiten comprender una cadena de acontecimientos que, mediados por sentimientos de insatisfacción, vulnerabilizan al(a la) trabajador(a) y lo(la) hacen sufrir. Este conjunto de factores potencia las ideas suicidas y le lleva a considerar el suicidio como la alternativa que pondrá fin a su sufrimiento.

## Introdução

O suicídio constitui um grave problema de saúde pública em escala mundial. De acordo com dados da Organização Mundial da Saúde (OMS), aproximadamente 800 mil pessoas morrem anualmente em decorrência desse fenômeno, o que equivale a uma morte a cada 40 segundos, com uma taxa global estimada em 11,6 por 100 mil habitantes ^1^. Entre adultos jovens, o suicídio representa a segunda principal causa de mortalidade ^1^. No Brasil, a situação é igualmente preocupante: em 2019, foram registrados 14.540 casos, posicionando o país como o oitavo no ranking mundial e o segundo nas Américas em números absolutos, correspondendo a uma ocorrência a cada 36 minutos ^1^ ^,^ ^2^. Entre 1996 e 2015, contabilizaram-se mais de 170 mil casos, evidenciando a magnitude do problema ao longo das últimas décadas. No Estado da Bahia, especificamente, entre 2010 e 2019 foram notificados 5.160 casos, dos quais 634 ocorreram apenas em 2019 ^3^.

Estabelecer a relação entre o suicídio e trabalho enfrenta a visão tradicional que atribui ao fenômeno algo subjetivo e do âmbito individual, estabelecendo inadequações particulares como as causas únicas de sua ocorrência. Os modos de degradação do tecido social, no qual o trabalho tem papel central e que produz o suicídio é, continuamente, invisibilizado e decorrem de processos sociais que isolam indivíduos considerados como não adaptados ^2^ ^,^ ^4^ ^,^ ^5^ ^,^ ^6^.

Como consequência desta exclusão, emergem sentimentos e sensações que se configuram como precursores do contexto suicida: solidão, pertencimento frustrado, sobrecarga percebida, incapacidade para o trabalho, desesperança, sentido de inutilidade, que estruturam percepções deturpadas de si mesmo e que se transformam em culpa. Insere-se neste emaranhado, as pressões por produtividade que têm na competitividade entre colegas de trabalho, o *modus operandi* que tensionam os afetos e geram os sofrimentos ^7^ ^,^ ^8^ ^,^ ^9^ ^,^ ^10^ ^,^ ^11^. O medo, muito presente, reforça a impressão do sentimento de fraqueza frente à rotina de trabalho, levando o indivíduo aos picos de sofrimento e adoecimento - caminho livre para o contexto suicida ^10^ ^,^ ^11^. Dejours & Bègue ^4^ destacam que, negar o sofrimento faz parte das estratégias de defesa coletivas, como medida de proteção ao desemprego, à retaliação pelo patronado e aos preconceitos associados aos transtornos mentais, que geram e alimentam estigmas e exclusão.

O suicídio não ocorre repentinamente. Em geral, o indivíduo apresenta sinais e sintomas que indicam a sua intenção. Há, portanto, um contexto que envolve características que sinalizam a situação. A ideação suicida ^12^ ^,^ ^13^, os planos ^14^ ^,^ ^15^ e tentativas prévias ^14^ ^,^ ^16^ ^,^ ^17^, materializam o sofrimento psíquico em estágio mais agudo. Outros eventos predominantemente identificados na literatura, associados a este contexto, compõem um corpo de indicadores que auxiliam na compreensão do fenômeno e nas ações para sua mitigação. 

Neste estudo, a insatisfação é apresentada como importante mediadora para a ideação suicida. Esta dimensão, apesar de seu caráter subjetivo, pode ser produzida por questões concretas do cotidiano, sendo neste estudo consideradas: a insatisfação com o trabalho ^7^ ^,^ ^8^, as relações sociais ^8^ ^,^ ^18^ ^,^ ^19^, qualidade de vida ^8^ ^,^ ^18^, com a saúde ^18^ ^,^ ^19^ e consigo próprio ^8^ ^,^ ^20^. Se conjugadas, tornam-se indicadores relevantes ao monitoramento de situações de vulnerabilidade e risco associado a este desfecho. 

Diante de um panorama de investigações centradas em características individualizadas dos sujeitos, algo tão complexo como o suicídio restringe-se, em geral, ao que é mensurável e que se esteja restrito à esfera individual. Perspectiva esta, insuficiente, uma vez que as condições elencadas não estão na gênese do problema, mas são produtos dele e funcionam como mediadores para o contexto suicida. Portanto, a busca pela compreensão dos processos que (re)produzem coletivamente o sofrimento necessita de relevo. Há de se ultrapassar a visão tradicional pontual e incluir análises que compreendam tal fenômeno como processual e dependente de fatores que (in)diretamente o define ^5^ ^,^ ^6^ ^,^ ^21^ ^,^ ^22^ ^,^ ^23^ ^,^ ^24^ ^,^ ^25^. 

Este estudo focaliza aspectos relacionados ao que foi intitulado de insatisfação global como precursor direto da ideação suicida em trabalhadores(as). Sendo esta característica mediadora de fatores associados ao sofrimento psíquico, destacando-se: estressores ocupacionais ^26^ ^,^ ^27^ ^,^ ^28^, sexo ^5^ ^,^ ^29^ ^,^ ^30^, baixa renda ^31^ ^,^ ^32^ ^,^ ^33^ ^,^ ^34^ e ausência de lazer ^21^. 

O contexto do suicídio, marcado por sua complexidade e gravidade, exige esforços abrangentes e coordenados diante do desafio que representa para a saúde pública. Objetiva-se, então, investigar a associação entre aspectos psicossociais do trabalho, fatores socioeconômicos e a insatisfação global com a ideação suicida em trabalhadores(as).

## Métodos

### Tipo de estudo

Estudo com dados pontuais no tempo oriundos da coorte prospectiva: *Vigilância em Saúde Mental e Trabalho: Uma Coorte da População de Feira de Santana-BA*, financiada pela Fundação de Amparo à Pesquisa do Estado da Bahia (FAPESB/SESAB/CNPQ/MS PPSUS/BA - 028/2018).

### População, amostra e critérios de elegibilidade

A população de estudo é constituída por indivíduos com 15 ou mais anos de idade, residentes em zonas urbanas do Município de Feira de Santana, Bahia, Brasil. O procedimento amostral levou em consideração cinco subdistritos do município, estabelecendo-se sorteio de conglomerados em dois estágios (setores censitários e ruas). Os sorteios em cada subdistrito obedeceram às etapas: amostragem simples das unidades primárias (setor censitário); em cada setor procedeu-se amostragem probabilística proporcional das ruas, considerando a proporção domicílios/rua. Utilizou-se dados do Instituto Brasileiro de Geografia e Estatística (IBGE) ^35^. 

O cálculo do tamanho amostral mínimo foi determinado considerando o sexo como exposição principal. Os parâmetros incluíram a prevalência de transtornos mentais comuns de 26% entre os não expostos positivos (homens) e 35,9% nos expostos positivos (mulheres) (dados da linha de base - 2007 ^36^). Estabeleceu-se um nível de precisão de 95% de confiança, 90% de poder de detecção, razão de risco de 1,38, diferença de risco entre os grupos de 10,0 e efeito de desenho de 2. Com base nisso, a amostra inicial estimada foi de 2.212 indivíduos. Adicionalmente, foram incluídos 20% para perdas, resultando em uma amostra mínima total de 2.655 participantes (1.793 mulheres e 862 homens). Como a ideação suicida (desfecho deste estudo), possui baixa prevalência, o cálculo amostral realizado para o estudo maior tem o poder de detecção das associações pretendidas.

Foram adotados como critérios de inclusão: idade igual ou superior a 15 anos; e residir no domicílio. Foram excluídos indivíduos: que trabalhassem ou dormissem no domicílio, mas não residissem no mesmo; que possuíssem algum tipo de deficiência ou sofrimento mental que inviabilizasse responder verbalmente ao questionamento oral.

### Procedimentos de coleta de dados

A coleta de dados ocorreu entre dezembro de 2018 a novembro de 2019. Foram realizadas entrevistas individuais, face a face, no domicílio, por equipe de pesquisa previamente treinada, respeitando sua privacidade. Para reduzir as perdas e entrevistar cada participante elegível do domicílio, os pesquisadores realizaram até três visitas ao local, em dias e turnos alternados. Somente após esgotadas as tentativas considerou-se perda.

### Instrumento de coleta de dados e variáveis do estudo

Para este estudo, foram extraídas variáveis dos dez blocos do questionário utilizado na coorte, a saber.

(a) Características sociodemográficas: sexo (0: masculino; 1: feminino) e renda mensal (dicotomizada pela mediana em 0: até R$ 1.000,00; 1: R$ 1.000,00 ou mais);

(b) Características ocupacionais: situação de trabalho (0: trabalha; 1: não trabalha) e os estressores ocupacionais: comprometimento excessivo com o trabalho e demanda emocional do trabalho.

O comprometimento excessivo com o trabalho foi avaliado pelo *Effort-Reward Imbalance* (ERI), validado para uso no Brasil ^37^ ^,^ ^38^ ^,^ ^39^. Essa dimensão foi mensurada por seis itens com respostas em escala Likert, indo de 1 (discordo totalmente) a 4 (concordo totalmente). Após a soma das pontuações atribuídas a cada item, aplicou-se o ponto de corte maior ou igual ao terceiro tercil para classificar a ausência ou presença do comprometimento excessivo com o trabalho. 

A demanda emocional do trabalho foi avaliada pelo *Job Content Questionnaire* (JCQ), utilizando-se três itens “meu trabalho exige muito emocionalmente”, “meu trabalho envolve negociação/conversa/entendimento com outras pessoas” e “em meu trabalho preciso suprimir minhas verdadeiras emoções”, com opções de resposta em escala Likert que variaram de 1 (discordo) a 4 (concordo fortemente). O escore, obtido pelo somatório dos itens, foi dicotomizado em nível baixo e alto (exposição), utilizando-se o terceiro tercil como ponto de corte.

(c) Prática de atividade de lazer (0: não; 1: sim). Foram consideradas como lazer as atividades: jogos, cinema/teatro, festa/seresta, barzinho, praia/piscina, viagens, igreja, TV/rádio/leitura. Os participantes, após avaliarem essas atividades, responderam ao questionamento de praticar ou não atividades de lazer. Na modelagem com equações estruturais, optou-se por designar a variável como “ausência de atividades de lazer” e categorizá-la em 0 (não) e 1 (sim).

(d) Ideação suicida: aplicou-se o *Self-Reporting Questionnaire* (SRQ-20), escala validada para uso em trabalhadores no Brasil ^40^, que avalia a presença de sintomas ansiosos/somáticos/depressivos dos últimos 30 dias. Para a ideação suicida, analisou-se a questão 17: “tem tido ideia de acabar com a vida?” (0: não; 1: sim).

(e) Insatisfação global: foram utilizadas questões do *World Health Organization Quality of Life* (WHOQOL-BREF) ^41^ para avaliar: satisfação com a capacidade para o trabalho, relações pessoais, consigo mesmo e qualidade de vida. Foi avaliada, também, a satisfação com a saúde. Os itens foram dicotomizados (0: satisfeito(a); 1: insatisfeito(a)).

### Modelo estrutural e hipóteses de estudo

**Figura 1:**
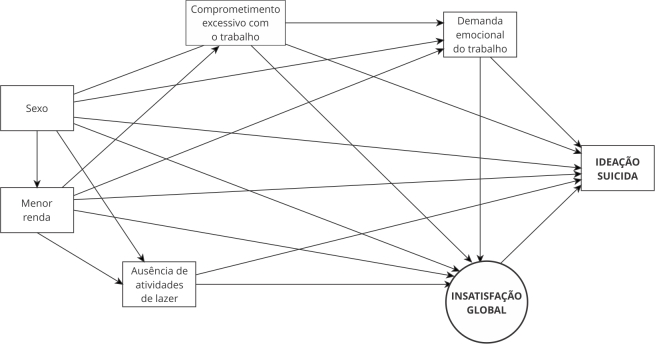
Modelo estrutural de determinantes da ideação suicida em trabalhadores(as).

Foi elaborado um modelo estrutural de determinantes da ideação suicida em trabalhadores(as) (Figura 1). A hipótese principal é que a insatisfação global tem efeito direto sobre a ideação suicida e é mediadora do efeito indireto dos demais fatores (sexo, renda mensal, prática de atividades de lazer, comprometimento excessivo com o trabalho e demanda emocional do trabalho).

Fonte: elaboração própria.

### Tratamento e análise dos dados

Sistematizou-se banco de dados no software SPSS (https://www.ibm.com/). Para as variáveis explanatórias do modelo estrutural, foram estimadas frequências absolutas e relativas da amostra. Em seguida, foram estimadas prevalências da ideação suicida (intervalo de 95% de confiança - IC95%), de acordo com as variáveis explanatórias e realizou-se análise bivariada, por meio do teste qui-quadrado de Pearson com significância estatística de 5%. Por fim, a modelagem de equações estruturais foi processada utilizando-se o software Mplus (https://www.statmodel.com/), aplicando-se a ponderação pelos pesos amostrais, com estratificação por subdistritos e *clusters* de setores censitários.

Para condução da modelagem de equações estrutirais, foi realizada a mensuração do construto latente insatisfação global ^42^. Conduziu-se a avaliação da estrutura fatorial das variáveis diretamente observadas por meio da análise fatorial exploratória (AFE), onde o número de fatores extraídos foi determinado por autovalores ≥ 1 ^43^. A unidimensionalidade do construto foi validada por meio de análise fatorial confirmatória (AFC). O critério para retenção do item no fator foi apresentar carga fatorial padronizada ≥ 0,3 e variância residual ≤ 0,7 ^44^ ^,^ ^45^.

O modelo estrutural foi constituído por insatisfação global (exposição principal), pelas variáveis observadas (sexo, renda mensal, prática de atividades de lazer e estresse ocupacional) e a variável resposta: ideação suicida. Por meio de AFC, foram estimados os coeficientes de regressão (β) padronizados e respectivos valores de p (α = 5%). Os efeitos diretos, indiretos e totais foram classificados quanto ao tamanho em: pequeno (por volta de 0,10), médio (em torno de 0,30) e grande (cerca de 0,50 ou mais) ^46^.

Para as análises fatoriais foi utilizado o estimador média dos mínimos quadrados ponderados e variância ajustada (WLSMV, acrônimo em inglês). Foram inspecionados os índices de modificação (MI, acrônimo em inglês, ≥ 10) e as mudanças de parâmetros esperadas (EPC, acrônimo em inglês, ≥ 0,25) para verificar a necessidade de reespecificar o modelo ^47^. Para avaliar a qualidade do ajuste dos modelos foi utilizado o qui-quadrado normalizado (χ^2^/graus de liberdade - g.l. < 2) ^48^; o erro quadrático médio da aproximação (RMSEA, acrônimo em inglês, < 0,06), com respectivo IC90% < 0,08 ^47^; o índice de ajuste comparativo (CFI, acrônimo em inglês, ≥ 0,95) e o índice de Tucker-Lewis (TLI, acrônimo em inglês, ≥ 0,95) ^46^. 

### Aspectos éticos e legais

O estudo foi aprovado pelo Comitê de Ética em Pesquisa da Universidade Estadual de Feira de Santana (protocolo nº 2.420.653), concernente às resoluções éticas do Conselho Nacional de Saúde do Brasil, respeitando-se seus princípios de equidade, justiça, benevolência, não maleficência e respeito a autonomia, manifestada voluntariamente a partir dos termos de consentimento e assentimento. As entrevistas foram procedidas respeitando a privacidade de cada participante.

## Resultados

Participaram do estudo 1.629 trabalhadores(as). Predominaram mulheres (892; 54,8%); renda mensal de R$ 1.000,00 ou mais (521; 62%); e, que alegaram gozarem de atividades de lazer (1.336; 82,6%). Com relação às características ocupacionais, houve maior proporção de pessoas sem comprometimento excessivo (986; 66,6%) e com baixa demanda emocional (1.305; 86,8%). Ao se observar os níveis de insatisfação, predominaram satisfeitos com a capacidade para o trabalho (1.302; 86,3%), satisfeitos com as relações pessoais (1.431; 88,4%), satisfeitos consigo mesmo (1.403; 86,7%), com a qualidade de vida (1.139; 70,4%) e com percepção positiva sobre a própria saúde (1.153; 71,3%) (Tabela 1).

**Tabela 1:** Prevalência de ideação suicida em trabalhadores(as), segundo características sociodemográficas, prática de lazer, estressores ocupacionais e insatisfação. Feira de Santana, Bahia, Brasil, 2019 (N = 1.629).

Variáveis	Amostra			Ideação suicida						Valor de p
				Não			Sim			
	n	%	IC95%	n	%	IC95%	n	%	IC95%	
Sexo										< 0,001
Masculino	737	45,2	42,8-47,7	711	96,9	95,3-98,0	23	3,1	2,0-4,7	
Feminino	892	54,8	52,3-57,2	820	92,0	90,0-93,7	71	8,0	6,3-9,9	
Renda mensal (n = 841)										0,053
Até R$ 1.000,00	320	38,0	34,8-41,4	294	92,2	88,6-94,9	25	7,8	5,1-11,4	
R$ 1.000,00 ou mais	521	62,0	58,6-65,2	497	95,4	93,2-97,0	24	4,6	3,0-6,8	
Ausência de atividades de lazer (n = 1.618)										0,002
Não	1.336	82,6	80,6-84,4	1.268	95,0	93,7-96,2	66	5,0	3,8-6,3	
Sim	282	17,4	15,6-19,4	253	90,4	86,3-93,5	27	9,6	6,5-13,7	
Comprometimento excessivo com o trabalho (n = 1.480)										< 0,001
Ausente	986	66,6	64,2-69,0	948	96,3	95,0-97,4	36	3,7	2,6-5,0	
Presente	494	33,4	31,0-35,8	444	90,1	87,1-92,3	49	9,9	7,4-12,9	
Demanda emocional do trabalho (n = 1.503)										0,001
Baixa	1.305	86,8	85,0-88,5	1.238	95,1	93,8-96,2	64	4,9	3,8-6,2	
Alta	198	13,2	11,5-15,0	177	89,4	84,2-93,3	21	10,6	6,7-15,8	
Insatisfação com capacidade para o trabalho (n = 1.509)										0,001
Satisfeito	1.302	86,3	84,4-88,0	1.237	95,2	93,8-96,3	63	4,8	3,7-6,2	
Insatisfeito	207	13,7	12,0-15,6	184	89,3	84,3-93,2	22	10,7	6,8-15,7	
Insatisfação com as relações pessoais (n = 1.618)										< 0,001
Satisfeito	1.431	88,4	83,9-91,7	1.368	95,7	94,5-96,7	62	4,3	3,3-5,5	
Insatisfeito	187	11,6	8,3-16,1	153	82,7	74,5-87,9	32	17,3	12,1-23,5	
Insatisfação consigo mesmo (n = 1.619)										< 0,001
Satisfeito	1.403	86,7	86,8-90,0	1.347	96,2	95,0-97,1	54	3,8	2,9-5,0	
Insatisfeito	216	13,3	11,7-15,1	174	81,3	75,4-86,3	40	18,7	13,7-24,6	
Insatisfação com a qualidade de vida (n = 1.618)										< 0,001
Satisfeito	1.139	70,4	68,1-72,6	1.101	96,8	95,5-97,7	37	3,2	2,3-4,5	
Insatisfeito	479	29,6	27,2-31,7	420	88,0	84,8-90,8	57	12,0	9,2-15,2	
Insatisfação com a saúde (n = 1.617)										< 0,001
Positiva	1.153	71,3	69,0-73,5	1.108	96,3	95,0-97,3	43	3,7	2,7-5,0	
Negativa	464	28,7	26,5-31,0	413	89,2	86,0-91,9	50	10,8	8,1-14,0	

IC95%: intervalo de 95% de confiança.

Fonte: elaboração própria.

A prevalência de ideação suicida entre os(as) trabalhadores(as) foi de 5% (n = 94/1.625; IC95%: 3,3-7,6). Houve predomínio da ideação suicida em mulheres (8%), menor renda (7,8%), ausência de atividades de lazer (9,6%), com comprometimento excessivo com o trabalho (9,9%), alto nível de demanda emocional do trabalho (10,6%), insatisfação com a capacidade para o trabalho (10,7%), com as relações pessoais (17,3%), consigo mesmo (18,7%), com a qualidade de vida (12%) e com a saúde (10,8%) (Tabela 1). As variáveis explanatórias apresentaram associação estatisticamente significante com a ideação suicida, com exceção da renda mensal (associação *borderline*).

O modelo de mensuração da variável latente (insatisfação global) obteve índices de ajuste satisfatórios (AFE: χ^2^/g.l. = 1,5; RMSEA = 0,029; IC90%: 0,017-0,042; CFI = 0,989; TLI = 0,978; AFC: χ^2^/g.l. = 1,5; RMSEA = 0,017; IC90%: 0,000-0,042; CFI = 0,989; TLI = 0,978. A presença de apenas um autovalor revelou a estrutura unidimensional de insatisfação global. Na AFE, todas as cargas fatoriais do modelo de mensuração foram fortes e estatisticamente significantes. Não foram sugeridas correlações residuais pelos índices de modificação de efeito (Tabela 2).

**Tabela 2:** Cargas fatoriais padronizadas do modelo de mensuração de insatisfação global de trabalhadores(as). Feira de Santana, Bahia, Brasil, 2019 (N = 1.629).

Variáveis indicadoras	AFE				AFC	
	Autovalores		λ	Valor de p	λ	Valor de p
	1	2				
	2,72	0,85				
Insatisfação com capacidade para o trabalho			0,575	≤ 0,05	0,631	< 0,001
Insatisfação com as relações pessoais			0,815	≤ 0,05	0,756	< 0,001
Insatisfação consigo mesmo			0,877	≤ 0,05	0,845	< 0,001
Insatisfação com a qualidade de vida			0,597	≤ 0,05	0,479	< 0,001
Insatisfação com a saúde			0,420	≤ 0,05	0,372	< 0,001

λ: cargas fatoriais padronizadas; AFC: análise fatorial confirmatória; AFE: análise fatorial exploratória.

Fonte: elaboração própria.

**Figura 2:**
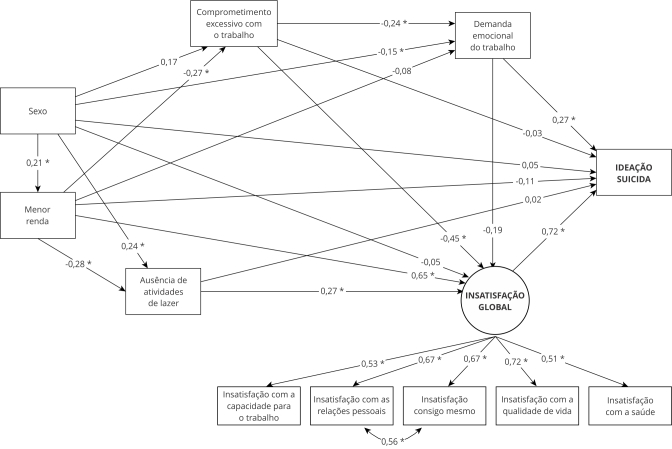
Modelo de equações estruturais com efeitos diretos e indiretos específicos para a ideação suicida em trabalhadores(as). Feira de Santana, Bahia, Brasil, 2019 (N = 1.629).

No modelo estrutural, a variável resposta foi a ideação suicida e as variáveis explanatórias: a latente (insatisfação global) e as variáveis: sexo, menor renda mensal, ausência de atividades de lazer, comprometimento excessivo e demanda emocional do trabalho (Figura 2). Os índices de ajuste do modelo estrutural foram satisfatórios: χ^2^/g.l. = 1,4; RMSEA = 0,015; IC90%: 0,000-0,025; CFI = 0,955; TLI = 0,917.

Fonte: elaboração própria.

O fator que mais contribui para a ideação suicida em trabalhadores(as) foi a insatisfação global. Identificou-se efeito direto grande (β = 0,72; valor de p = 0,001) e médio para demanda emocional do trabalho (β = 0,27; valor de p = 0,007). Esses resultados evidenciam que, além da insatisfação global, a alta demanda emocional do trabalho incrementam, diretamente a prevalência de ideação suicida (Figura 2). 

Além dos efeitos diretos observados, foram identificados efeitos indiretos específicos, significativamente associados a ideação suicida: (a) mulheres que não praticavam atividades de lazer (β = 0,24; valor de p = 0,001), maior nível de insatisfação global (β = 0,27; valor de p < 0,001) e maior prevalência de ideação suicida (β = 0,21; valor de p = 0,005); (b) mulheres com menor renda mensal (β = -0,21; valor de p < 0,001), maior insatisfação global (β = 0,65; valor de p < 0,001) e maior prevalência de ideação suicida; (c) mulheres com alta demanda emocional do trabalho (β = 0,15; valor de p = 0,042) apresentaram maior ocorrência de ideação suicida; (d) o efeito direto de renda mensal não foi significante, contudo, verificou-se que, quanto menor a renda mensal do(a) trabalhador(a) maior a insatisfação global (β = 0,65; valor de p < 0,001) e maior a ideação suicida; (e) maior renda associou-se à ausência de atividades de lazer (β = -0,28; valor de p = 0,033), maior nível de insatisfação global e maior ideação suicida; (f) comprometimento excessivo com o trabalho não teve efeito direto significante sobre a ideação suicida, mas sua presença aumentou a demanda emocional do trabalho (β = 0,24; valor de p = 0,013) e ideação suicida; (g) o comprometimento excessivo com o trabalho associou-se a maior nível de insatisfação global (β = 0,45; valor de p < 0,001); (h) trabalhadores(as) que não praticavam atividades de lazer possuíam maior insatisfação global (β = 0,27; valor de p < 0,001) e ideação suicida; (i) ausência de lazer não apresentou efeito direto significante, porém, sua ausência associou-se à maior insatisfação global (β = 0,27; valor de p < 0,001), resultando em maior ideação suicida (Figura 2).

A demanda emocional no trabalho apresentou efeito sobre a ideação suicida. O comprometimento excessivo com o trabalho, apesar de não apresentar efeito direto, apresentou efeito total significante (λ = 0,319; valor de p < 0,001). Houve também efeito total significante de menor renda mensal (λ = 0,207; valor de p = 0,006) e *borderline* para não praticar atividade de lazer (λ = 0,216; valor de p = 0,056), com tamanho de efeito médio. Houve efeito total pequeno para sexo, com maior exposição entre as mulheres (λ = 0,177; valor de p = 0,008) (Tabela 3). 

**Tabela 3:** Efeitos totais e indiretos específicos padronizados do modelo de equações estruturais de fatores determinantes da ideação suicida em trabalhadores(as). Feira de Santana, Bahia, Brasil, 2019 (N = 1.629).

Caminhos	Coeficiente padronizado	Valor de p
Efeitos totais		
SEX→IS	0,177	0,008 *
MREN→IS	0,207	0,050 *
AULAZ→IS	0,216	0,056 **
COMP→IS	0,319	0,000 *
DEMO→IS	0,130	0,203
Efeitos indiretos específicos		
SEX		
SEX→MREN→IS	-0,023	0,645
SEX→MREN→INSAT→IS	0,100	0,065
SEX→MREN→AULAZ→IS	-0,001	0,889
SEX→MREN→AULAZ→INSAT→IS	-0,012	0,283
SEX→MREN→COMP→DEMO→IS	-0,004	0,230
SEX→MREN→COMP→DEMO→INSAT→IS	0,002	0,381
SEX→MREN→COMP→IS	0,002	0,831
SEX→MREN→COMP→INSAT→IS	-0,018	0,202
SEX→MREN→DEMO→IS	-0,005	0,320
SEX→MREN→DEMO→INSAT→IS	0,003	0,345
SEX→AULAZ→ INSAT→IS	0,048	0,187
SEX→AULAZ→IS	-0,006	0,889
SEX→COMP→IS	-0,006	0,853
SEX→COMP→DEMO→IS	0,011	0,238
SEX→COMP→DEMO→INSAT→IS	-0,006	0,383
SEX→COMP→INSAT→IS	0,055	0,202
SEX→DEMO→IS	0,042	0,138
SEX→DEMO→INSAT→IS	-0,022	0,273
SEX→INSAT→IS	-0,039	0,547
MREN		
MREN→DEMO→IS	-0,023	0,383
MREN→COMP→IS	0,009	0,835
MREN→AULAZ→IS	-0,006	0,887
MREN→INSAT→IS	0,470	0,034 *
MREN→COMP→DEMO→IS	-0,017	0,206
MREN→DEMO→INSAT→IS	0,012	0,403
MREN→COMP→INSAT→IS	-0,086	0,167
MREN→AULAZ→INSAT→IS	-0,054	0,327
MREN→COMP→DEMO→INSAT→IS	0,009	0,368
AULAZ		
AULAZ→INSAT→IS	0,196	0,114
COMP		
COMP→DEMO→IS	0,064	0,079
COMP →INSAT→IS	0,323	0,026 *
COMP →DEMO→INSAT→IS	-0,033	0,271
DEMO		
DEMO→INSAT→IS	-0,140	0,139

AULAZ: ausência de lazer; COMP: comprometimento excessivo com o trabalho; DEMO: demanda emocional do trabalho; INSAT: insatisfação global; IS: ideação suicida; MREN: menor renda mensal; SEX: sexo.

Fonte: elaboração própria.

* Valor de p ≤ 0.05;

** Associação *borderline*.

Destacaram-se sobre a ideação suicida, mediados pelo maior nível de insatisfação global: a menor renda mensal (λ = 0,470; valor de p = 0,034) e o comprometimento excessivo com o trabalho (λ = 0,323; valor de p = 0,026). A magnitude desses efeitos foi grande para menor renda e médio para comprometimento excessivo com o trabalho, com associação estatisticamente significante (Tabela 3).

## Discussão

Este estudo identificou que os aspectos psicossociais do trabalho e os fatores socioeconômicos estão associados a ideação suicida e são mediados pela insatisfação global.

A insatisfação é um produto de um modelo de (re)produção da vida. Portanto, tem conformação nas impressões sobre o trabalho e sobre a própria capacidade de trabalhar; sobre as relações sociais, qualidade de vida e o entendimento de si, seu corpo e subjetividade, que produzem sua saúde, implicando que condições crônicas/limitantes sejam indicadores que conformam a (in)satisfação global ^7^ ^,^ ^8^ ^,^ ^12^ ^,^ ^13^ ^,^ ^14^ ^,^ ^18^ ^,^ ^19^ ^,^ ^20^. Essas formas de produzir a vida são comuns aos trabalhadores do mundo, bem como os sofrimentos provenientes delas ^4^. 

A discussão sobre as ocupações específicas não foram aprofundadas neste estudo. Porém, os dados indicam situação de precarização: aproximadamente 40% dos trabalhadores recebiam menos de um salário (menos de R$ 1,000,00 mensais), o que caracteriza condição de trabalho precarizado - condição na qual as pessoas não estão tendo acesso a uma remuneração digna - isso produz sofrimentos e sensação de desvalorização social aos trabalhadores. Quando as condições materiais do trabalho são insuficientes para prover as necessidades da vida cotidiana, as demais dimensões da vida são afetadas ^2^ ^,^ ^5^ ^,^ ^17^ ^,^ ^18^ ^,^ ^21^. Esses sentimentos de desvalia social e pessoal decorrem de tensionamentos entre forças historicamente assimétricas: interesses do capital *versus* a luta das classes trabalhadoras. Como os interesses do capital quase sempre prevalecem, emergem as condições que geram sofrimentos ^49^ ^,^ ^50^.

Alguns estudos sugerem que dimensões de insatisfação, com gênese e caminhos distintos, mas também coincidentes, estão associadas a ideação suicida. Na China, o bem-estar é associado negativamente com a ideação suicida e, ações que caminhem neste sentido, fazem parte do escopo estrutural para o enfrentamento do problema ^27^ ^,^ ^28^. 

O caráter amplo da insatisfação global, transita em questões diversas que acometem o indivíduo e que provocam o sofrimento, como a angústia, o medo e a desesperança ^9^ ^,^ ^10^ ^,^ ^11^. Portanto, ela media eventos que produzem sofrimento, adoecimento e morte. Um fator que exemplifica este caminho, muito comum em produzir a insatisfação com a vida, com a própria condição para o trabalho e consigo próprio, é a presença de doenças crônicas de longa duração, que na literatura, estão amplamente associadas ao contexto suicida ^27^ ^,^ ^51^ ^,^ ^52^ ^,^ ^53^. As doenças, de maneira geral, são expressões clínicas individuais, sob o ponto de vista fisiopatológico, porém precisam ser compreendidas como processos mais amplos, como manifestações coletivas, que ocorrem por diversos motivos e que geram impactos como o medo e a desesperança ^9^ ^,^ ^10^ ^,^ ^11^. Portanto a compreensão dos mecanismos geradores da insatisfação global, estão imbrincadas no tecido em que se estabelece a vida cotidiana, com relevo ao mundo do trabalho.

O trabalho é considerado determinante no processo saúde-doença. O(A) trabalhador(a), submetido a um contexto laboral de muitas transformações e pressões, precisa adaptar-se a estes fluxos, em um movimento de resiliência constante. Este contínuo que exige sua metamorfose tem como consequência o adoecimento, cuja severidade depende das forças de resistências físicas e/ou psicológicas do indivíduo ou dos grupos no trabalho ^4^.

Este estudo demonstrou efeito total, da demanda emocional do trabalho, e efeito total indireto, do comprometimento excessivo, com a ideação suicida. O comprometimento excessivo foi mediado pela insatisfação global. Tal dado apresenta um caminho a ser melhor investigado e deve fomentar novas discussões acerca da formação de tecnologias de enfrentamento a serem utilizadas pela vigilância em saúde do(a) trabalhador(a).

A demanda emocional do trabalho é um importante estressor ocupacional, bastante relacionado às psicopatologias como síndrome de Burnout, depressão e, por consequência, ideação suicida. Os fatores determinantes para a alta demanda emocional incluem exigências de contatos no trabalho envolvendo pressão, diferenças de gênero, natureza do trabalho a ser desenvolvido, qualidade no apoio social no trabalho e demandas de produtividade ^54^ ^,^ ^55^. Aliada a ausência de apoio social, na família e/ou no trabalho, a demanda emocional potencializa os efeitos de sentimentos de inadequação que podem produzir o contexto suicida ^55^ ^,^ ^56^ ^,^ ^57^ ^,^ ^58^.

Outra dimensão dos estressores ocupacionais refere-se à necessidade de reciprocidade nas trocas nas relações profissionais. Seu desequilíbrio imprime sofrimento, ou seja, quando o esforço no trabalho é maior que recompensa recebida por ele, eclode o adoecimento. Neste contexto, Araújo et al. ^22^ indicam uma terceira dimensão, o comprometimento excessivo com o trabalho, intimamente relacionado ao esforço e que tem como objetivo alcançar a estima profissional e o reconhecimento, ou aprovação pelos chefes e pares. O comprometimento excessivo com o trabalho apresentou o maior efeito total com a ideação suicida, e efeito indireto específico, mediado pela insatisfação global. Este achado está amplamente sustentado em publicações mundiais. O esforço elevado foi fator preditivo para a ideação suicida em trabalhadores fabris chineses ^26^. Em outra pesquisa ^6^, a pontuação média de esforço, associado ao excesso de comprometimento e pouca recompensa, foi relacionada à ideação suicida em 2.216 trabalhadores. 

A compreensão do processo saúde/doença no trabalho requer aprofundamento sobre as diferenças de sexo e sua influência no adoecimento. Neste estudo, ser mulher apresentou efeito total, como também se associou indiretamente com a ideação suicida. Os efeitos indiretos do sexo, foram mediados de três formas: não praticar atividades de lazer, gerando maior insatisfação global e ideação suicida; mulheres com menor renda, gerando maior insatisfação global e ideação suicida; e, alta demanda emocional do trabalho e ideação suicida. Em pesquisa ^5^ com mais de 20 mil trabalhadores(as) franceses(as), as mulheres tiveram destaque enquanto mais vulneráveis para a ideação suicida. Essas diferenças foram demonstradas em pesquisas na China, com mais de 2 mil trabalhadores(as) ^29^. Em outra pesquisa ^30^, ao se associar o estresse familiar e laboral, ao fato de ser mulher, as chances de ideação suicida aumentaram em cinco vezes, demonstrando que a sobrecarga de trabalho no lar, ainda é um fator importante e que merece maior atenção, quando somatizado às circunstâncias laborais formais femininas. 

O alto estresse no trabalho, estava associado a conflitos familiares, potencializando a ideação suicida, em mulheres ^30^, sendo as atividades de lazer medidas protetivas importantes. Sua ausência estava associada a maior estresse ocupacional, maior insatisfação global e ocorrência de ideação suicida. Na China, a diminuição na recreação foi associada à ideação suicida em trabalhadores(as) da indústria petrolífera ^21^. Kuhn & Flanagan ^54^ indicam que as mulheres estão mais vulneráveis a experimentar a exaustão emocional no trabalho, manifestando sinais de despersonalização, cinismo e menor realização. É importante refletir as iniquidades sexuais no trabalho formal, flexionadas ao cotidiano feminino em uma sociedade que, ao mesmo tempo, condiciona a mulher à luta desigual por melhores condições de trabalho, renda e maior reconhecimento e, exige delas as funções impostas por uma sociedade machista e sexista, cristalizada na sobrecarga doméstica. Essa condição determina menor tempo destinado às atividades de lazer, de cuidado pessoal, com a saúde e a autoestima, protetivas para o adoecimento mental.

Dentre os achados deste estudo, a menor renda associou-se à ideação suicida, mediado pela insatisfação global. Em pesquisa realizada em 59 países em desenvolvimento, com aproximadamente 230 mil pessoas, a região africana apresentou a maior prevalência de ideação suicida e planejamento de suicídio, sendo baixa e média renda fortemente associadas ao desfecho estudado ^31^ ^,^ ^32^ ^,^ ^33^. 

As perdas econômicas e dívidas são condições associadas ao suicídio ^2^ ^,^ ^34^ ^,^ ^56^. Policiais brasileiros que cometeram suicídio tinham, como característica altamente prevalente, o endividamento e/ou comprometimento com empréstimos financeiros ^2^. Em culturas tradicionais de trabalhadores rurais, o fracasso econômico e o não pagamento de suas dívidas pode determinar o autoextermínio ^57^. O mesmo motivo, com trabalhadores rurais, foi apontado em outro estudo ^34^, que adicionou problemas de ordem emocional e familiar, como gatilhos para o suicídio. Apesar deste estudo não avaliar as categorias de trabalho, é importante salientar que tal problema é sócio-historicamente determinada em trabalhadores do campo, como explica Neves ^56^ e reflete um modelo de exploração e expropriação que repercute na compreensão do fenômeno em todas as esferas do trabalho.

Identificou-se também neste estudo que trabalhadores com menor renda apresentaram associação com a ausência de atividade lazer, com maior insatisfação global e com a ideação suicida, sugerindo a potência que a insatisfação exerce sobre o desfecho, bem como a possibilidade de que trabalhadores excessivamente comprometidos na busca por melhor renda, desconsidere o tempo para o lazer, implicando em insatisfação global, no sofrimento psíquico e na ideação suicida.

De todo modo, tais situações sugerem a necessidade de esforços para a compreensão desses estressores, enquanto fatores que, em conjunto, potencializam a intensidade da ideação suicida. O apoio social no trabalho, com demandas justas, com sistemas de prevenção à violência (principalmente em ambientes sexistas), são alguns dos fatores que despontaram neste estudo, como também identificadas em pesquisas internacionais ^26^ ^,^ ^58^. Ressalta-se, ainda, no Brasil, ocorreu redução das estruturas que protegem o(a) trabalhador(a), que cada vez mais têm seus vínculos fragilizados, salários reduzidos, pressões internas por qualidade e produtividade e, externa, pelo desemprego e desamparo social (via reformas trabalhista, previdenciária e pela proposta de reforma administrativa recentes). Na China, os índices de ideação suicida em trabalhadores(as) aumentaram, coincidindo com a redução de recursos que eram destinados para a estrutura protetiva interna destes(as) trabalhadores(as) ^21^. Na Coreia do Sul, após a privatização de uma empresa estatal, benefícios como seguro médico e pensões para aposentadoria foram extintos, bem como os setores de saúde ocupacional e de vigilância em saúde. Estes(as) trabalhadores apresentaram aumento significativo para a ideação suicida ^59^. No Brasil, em uma coorte com 100 milhões de brasileiros de baixa renda, o acesso direto a políticas de distribuição de renda reduziu o risco para o suicídio ^60^.

O modelo de produção da vida centrado em imposições reificadas na modernização das relações que perpetuam as formas de produzir mais valia, geram eventos que foram denominados de “suicidamento” ^61^ e “mortes por desespero” (*deaths of despair*) ^62^. Estes termos caracterizam o suicídio contemporâneo, não como um processo tradicionalmente de natureza subjetiva e individual, mas sim como um produto do modo de viver e (re)produzir a vida, em que o trabalhador é compelido a uma situação tal, que o autoextermínio se torna uma alternativa considerada e, em alguns casos, efetivamente executada ^56^ ^,^ ^61^ ^,^ ^62^.

### Limitações do estudo

A ideação suicida é um fenômeno em que sua assumpção gera estigmas sociais e, por isso, pode subestimar sua frequência. Seu rastreio necessita de formas que consigam ser mais sensíveis ao problema. Nesta pesquisa, presume-se que, tal dificuldade, também tenha ocorrido, uma vez que tal desfecho foi estimado de forma direta. 

Na literatura, não foram identificadas pesquisas em que a exposição principal (insatisfação global) mediasse as associações entre as covariáveis e a ideação suicida. Para este estudo, a sustentação teórica se estabelece nas referências que indicam as insatisfações como fator proximal ao contexto do suicídio. Avaliar saúde mental de forma quantitativa apresenta desafios significativos em termos de aproximação teórica, portanto a sua mensuração, como realizada, implica em limitações para seu dimensionamento, mesmo sendo através de um item de instrumento validado para a população estudada. Os autores entendem que os resultados, ainda assim, são significativos, uma vez que o evento de interesse gera estigmas e geralmente são ocultados em inquéritos como o realizado. Novas formas de mensuração deste desfecho precisam ser consideradas para próximos estudos.

## Conclusões

Identificou-se que a alta demanda emocional do trabalho e a insatisfação global repercutem diretamente na ideação suicida. O comprometimento excessivo no trabalho conforma, junto a demanda emocional, importantes estressores ocupacionais que explicam o desejo de se matar. Maiores níveis de insatisfação global apresentaram efeitos diretos significantes sobre a ideação suicida em trabalhadores(as). O modelo explicativo apresenta, além dos estressores ocupacionais, diferenças de sexo, ausência de atividades de lazer, menor renda mensal e insatisfação global, amplamente sustentadas na literatura, quando associadas a desfechos de sofrimento mental.

Entender o suicídio no trabalho requer compreender que este fenômeno ocorre nos meios mais distintos de produção e não são exclusivamente atribuídas a uma ou outra condição. Têm-se uma conjuntura complexa e emergencial, no que tangem a (re)engenharia dos modos de produção e o (re)posicionamento coletivo, estabelecidos em função e finalidade dos seus atores na construção do trabalho.

Compreender a ideação suicida em grupos de trabalhadores(as) é uma tarefa que requer um nível elevado de esforços, uma vez que, pela natureza estigmatizadora, seu reconhecimento é silenciado e sua invisibilidade repercute, tanto nas estatísticas, quanto nas ações transformadoras necessárias ao mundo do trabalho e que permanecem incipientes. Políticas que permitam o rastreio efetivo, que incidam sobre a organização do trabalho, o adaptando às necessidades do(a) trabalhador(a), não o inverso, são condições mínimas, essenciais, para dirimir o contexto que gera sofrimento e morte. Instrumentos que consolidem a organização do trabalho com condições em que se inscrevem o contexto suicida de trabalhadores(as), ainda é um desafio que precisa ser superado.

Ainda assim, tais condições se enquadram em um panorama secundário de enfrentamento do problema, uma vez que, o processo de determinação social do suicídio, numa sociedade capitalista, nasce no trabalho e perpassa seus limites estruturais, permeando todos os campos da vida. Por ter acessibilidade complexa, a ideação suicida transita na invisibilidade e segue atenuada nas pormenorizações naturalizadas na perspectiva do limite aceitável do problema.
